# Modeling fertile window differences across the reproductive lifespan with quantitative urine hormone monitoring of the menstrual cycle

**DOI:** 10.1186/s13048-026-02184-w

**Published:** 2026-07-13

**Authors:** Thomas P. Bouchard, Richard J. Fehring, Maria Meyers, Theresa Stujenske, Louise Boychuk, Amanda Smith, Katherine Schweinsberg, Bruno Scarpa, Mary Schneider

**Affiliations:** 1 Department of Family Medicine, University of Calgary, 108-30 Springborough Blvd SW, Calgary, AB T3H 0N9 Canada; 2 College of Nursing, Marquette University, Milwaukee, USA; 3 Marquette Method Professional Association, Milwaukee, USA; 4 School of Nursing, Duquesne University, Pittsburgh, USA; 5 Department of Statistical Sciences, University of Padua, Padua, Italy; 6 University of British Columbia, Vancouver, Canada; 7 University of Calgary, Calgary, Canada

**Keywords:** Menstrual Cycle, Ovulation, Estrone-3-glucuronide (E_1_3G), luteinizing hormone (LH), Pregnanediol glucuronide (PDG)

## Abstract

**Background:**

Reproductive hormones of the fertile window are often referenced to women in regular cycles, but this may not be representative of the hormonal profiles of women in different circumstances like polycystic ovarian syndrome, the postpartum period, and the perimenopause transition. This observational cohort study sought to identify the variability in the reproductive hormones in various clinical circumstances and to establish potential thresholds for each category based on hormone measurements with the Mira urinary hormone monitor.

**Results:**

A total of 57 women (ages 22–51) in various circumstances (regular cycles, polycystic ovarian syndrome, postpartum and perimenopause) tracked Mira urine hormone measurements (estrone-3-glucuronide, luteinizing hormone, pregnanediol glucuronide), contributing 444 cycles of data. Using additive mixed models, hormone values were stratified by the four different reproductive categories. The perimenopause and polycystic ovarian syndrome groups demonstrated relative hypoestrogenic states, while the perimenopause group showed low luteal pregnanediol glucuronide and the polycystic ovarian syndrome/polyendocrine metabolic ovarian syndrome (PCOS/PMOS) group showed high luteal pregnanediol glucuronide. The perimenopause group had significantly higher luteinizing hormone values throughout the whole cycle.

**Conclusion:**

The fertile window hormone thresholds vary depending on a woman’s specific reproductive category. Women in different circumstances should not necessarily use the same hormonal thresholds for the fertile window and ovulation. A larger dataset with ultrasound correlation to ovulation is required to delineate the fertile window with more precision. Hormone differences across the menstrual cycle could be used for targeted treatments in polycystic ovarian syndrome and perimenopause women.

## Background

The Femtech industry, which includes wearables and personal devices to track the menstrual cycle, is rapidly expanding [1, 2]. The marketing of these technologies for menstrual cycle monitoring is outpacing evidence-based validation studies [3]. To expand the utility of these technologies for menstrual health monitoring, there is a need to examine their ability to detect menstrual health parameters in women at various reproductive stages [4, 5] and with common menstrual-related disorders, including polycystic ovarian syndrome (PCOS) recently renamed polyendocrine metabolic ovarian syndrome (PMOS) [6]. Menstrual cycle parameters are considerably variable [7], and should be assessed prospectively with accurate assessment approaches [3, 8]. The aim of the present study was to examine menstrual cycle parameters using self-tracked urinary reproductive hormone levels in postpartum and perimenopause women as well as those with PMOS.

Urinary hormone monitoring provides a non-invasive means of assessing underlying ovulatory function and menstrual cycle physiology [9]. The Mira monitor measures the urinary metabolites of follicle-stimulating hormone (FSH), estradiol in the form of estrone-3-glucuronide (E_1_3G), luteinizing hormone (LH), and progesterone in the form of pregnanediol glucuronide (PDG) previously described in detail [10]. Mira E_1_3G has been compared to serum estradiol in a recent study [11] and has now been robustly validated based on ultrasound and serum hormones [7, 12]. In the follicular phase of the menstrual cycle, rising levels of estrogen from the dominant follicle triggers a surge of luteinizing hormone from the anterior pituitary gland. This LH surge typically occurs 24–48 h before ovulation, leading to the release of a mature egg [13]. Following ovulation, the luteinized follicle (corpus luteum) produces progesterone, which is essential for preparing the endometrium for potential implantation [14]. Urine and serum reproductive hormone measurements demonstrate strong agreement [15], but unlike serum testing, which provides a measure of reproductive hormone levels at a single time point, daily first morning urine hormone testing enables a real-time analysis of hormone levels across the entire menstrual cycle. This type of hormonal testing could refine our understanding of menstrual cycle parameters, including follicular and luteal phase length, the fertile window, and timing of ovulation [10, 16–18]. The Mira monitor was chosen for the current study based on pilot data demonstrating good acceptability and ease of use by participants [10].

Tracking the fertile window is challenging in women with PMOS. The diagnosis of PMOS is based on meeting two of the following three consensus-based Rotterdam criteria [19]: irregular cycles, clinical and/or biochemical signs of hyperandrogenism, and polycystic ovaries on ultrasound. With the cycle irregularities of PMOS, related to estradiol dysregulation [20] and abnormal gonadotropin LH secretion [21], the Mira monitor has the potential to provide clarity to women with PMOS regarding their hormone patterns. It may also provide a way to track the hormonal effects of pre- and post-treatment interventions for PMOS.

Another challenging time to navigate the menstrual cycle is during postpartum amenorrhea and the first few transition cycles that are longer than usual [22]. The ClearBlue Fertility Monitor (CBFM) has been used in combination with the Marquette Method of family planning to track the return of fertility postpartum [23, 24], but does not provide quantitative hormone data to pinpoint the day of ovulation [25]. Using the Mira monitor to track E_1_3G, LH and PDG can help to identify which rises in E_1_3G and LH may potentially be ovulatory, with rising PDG to confirm the first ovulation postpartum. This would inform the postpartum return of fertility [4].

Finally, the perimenopause transition also presents difficulties related to irregular cycles, with variable hormonal patterns and disturbing vasomotor symptoms due to these hormonal changes [26]. This transition has been categorized in detail based on the Stages of Reproductive Aging Workshop (STRAW) in 2012 [27]. Instead of using a woman’s age as a marker for infertility, this system considers cycle length variability to describe reproductive stage and fertility. However, rather than just identifying cycle length variability, the Mira monitor may help to better delineate the hormonal transition as proposed in a recent case series [5].

In the current study, quantitative urine hormones (E_1_3G, LH and PDG; due to insufficient FSH data, FSH was not included), referenced to the LH surge day (the estimated day of ovulation) and confirmed by a rise in PDG, were compared between four reproductive categories to outline group differences in reproductive hormones during the fertile window and phases of the menstrual cycle.

## Methods

### Aim, design and setting

The aim of the present study was to assess whether or not significant differences in urine hormone profiles exist between women in different reproductive categories. The primary objective was to determine the feasibility of evaluating these differences in a larger sample of participants for future studies.

**Fig. 1:**
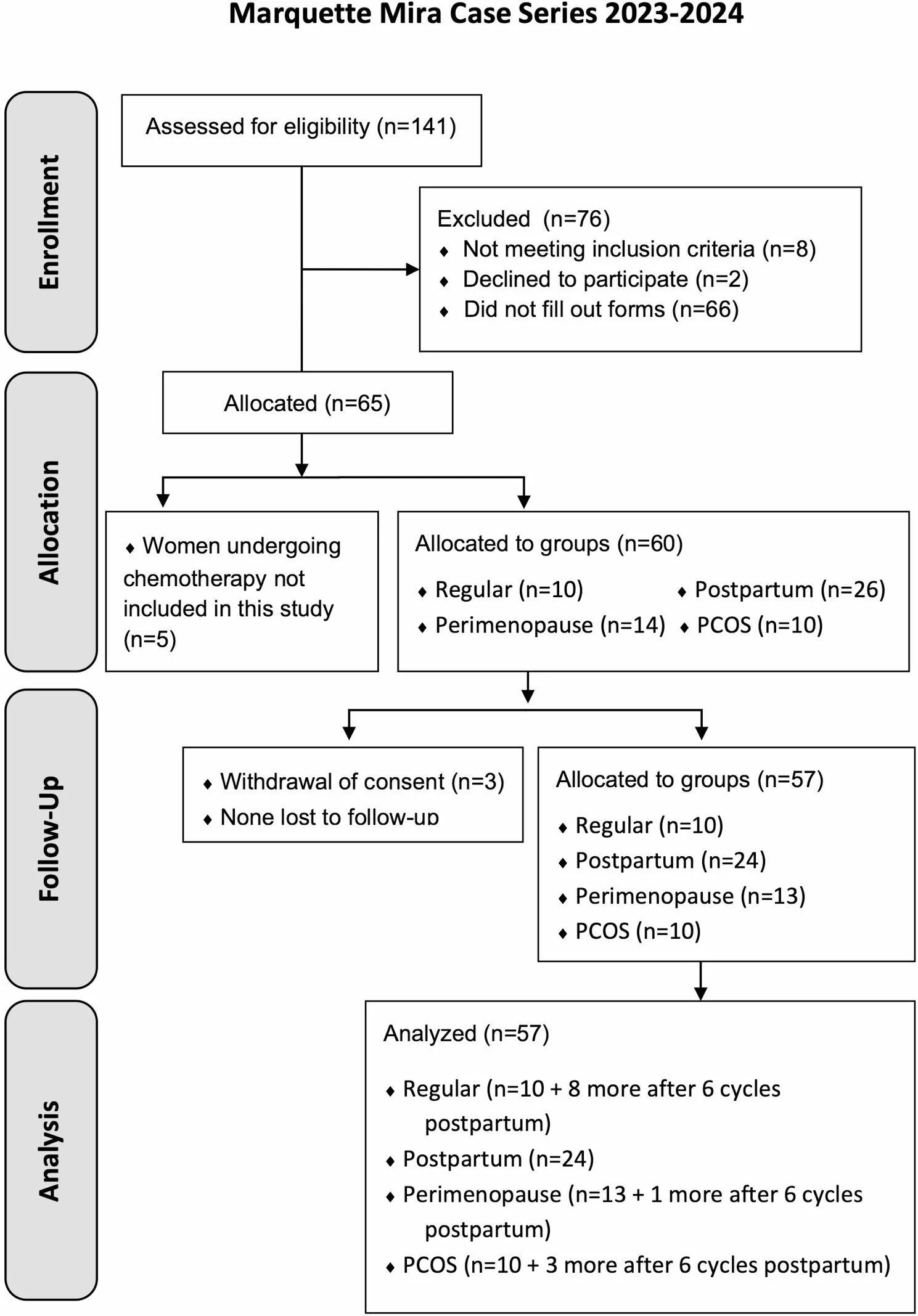
Consort Flow Diagram. Consort describing outlining initial enrollment, allocation to four groups, attrition (follow-up) and final analysis (*n*=57) with the breakdown of different groups. For women beyond 6 cycles postpartum, they were allocated into regular cycles (*n*=8), perimenopause (*n*=1) and PMOS (*n*=3)

This was an observational cohort study with prospective data collection (Fig. 1) using the Mira Monitor in various circumstances, with ethical approval by the Marquette University research ethics board (HR 4276, April 4, 2023) in accordance with the Declaration of Helsinki. Quanovate Tech (developers of the Mira monitor) were not involved in planning, interpretation, or review of the data or this manuscript. Inclusion criteria were: (1) English-speaking women between the ages of 18 and 52, (2) using their own Mira monitor and test wands, in (3) the four reproductive categories (regular cycles, PMOS, postpartum and perimenopause). Women were excluded if they: (1) were on hormonal medications that would impact ovulation, and (2) had a previous hysterectomy or oophorectomy. All participants provided informed consent to participate.

Recruitment was initiated via email to Marquette Method health care providers who teach women how to monitor their menstrual cycles. Eligible participants were asked to email the principal investigator (MS), who sent them a description of the study and links to complete: a consent form, a demographics form, and a chart data form. Participants consented to share their hormone data from an online Mira portal for health care providers. Forms and chart data were uploaded to a secure, private Marquette University OneDrive folder. Data was collected over 1 year, after which access to the portal was discontinued.

### Definitions of reproductive categories

The definition of regular cycles was based on previously established criteria [28], with consistent cycles (≤ 7 days variation between cycles) that were 24–38 days in length. Postpartum women were defined as being within delivery of a child in the last year and within the first 6 transition cycles postpartum after the initial postpartum amenorrhea (i.e. after postpartum amenorrhea, the first menses postpartum would be Cycle 1 and then the following 6 cycles). Postpartum women self-reported their breastfeeding status. The diagnosis of PMOS was self-reported but they had to confirm diagnosis by a clinician. Perimenopause participants were diagnosed based on the Stages of Reproductive Aging Workshop (STRAW) criteria [27] and were classified as early (> 7 day differences between cycle lengths), or late (there are > 60 days of amenorrhea).

### Menstrual cycle monitoring with urinary hormones

**Fig. 2:**
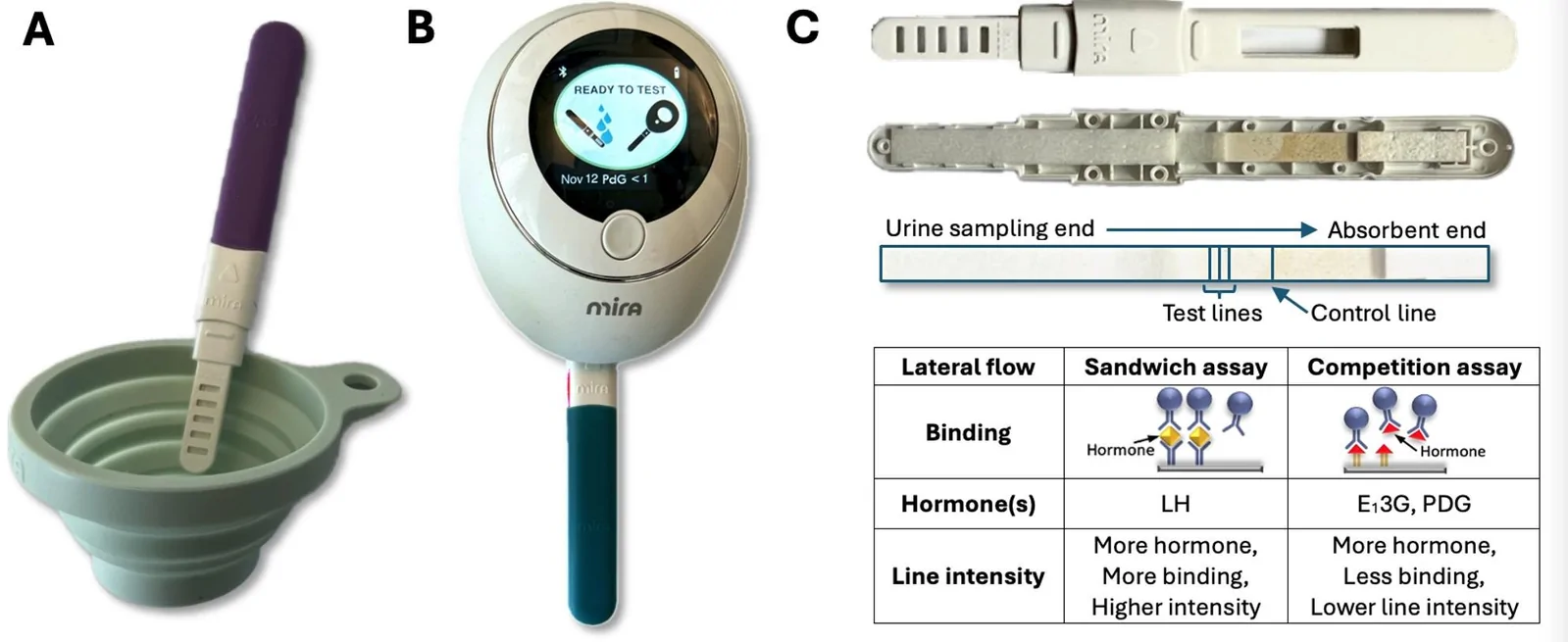
Mira Monitor testing and lateral flow assay. **A** Mira test stick dipped into first morning urine cup. **B** Insertion of test stick into the Mira monitor with optical analyzer. **C** Lateral flow assay view inside test stick with absorbent end on right and urine sampling end on left. The lateral flow assay is described based on hormone binding for sandwich and competition assays and how these affect line intensity

Participants tracked their menses, cycle length and used the Mira Monitor to test hormones in their first morning urine. Participants could use a Mira test wand that measures three hormone metabolites (E_1_3G, LH and PDG on the “Max” wand), or two test wands measuring the hormones separately (E_1_3G, LH on the “Plus” wand, and PDG on the “Confirm” wand). The wands are lateral flow assays using double antibody fluorescent labelling detected by the optical receiver in the monitor. The E_1_3G and PDG use a competition antibody assay, and the LH uses a sandwich assay (Fig. 2), previously described in detail [3].

### Definition of the estimated day of ovulation (EDO)

Preliminary results and pilot studies [10] have suggested that the Mira LH surge happens the day before or the day of ovulation. Based on calculations in comparison to the ClearBlue Fertility Monitor LH surge [10], which has been validated by ultrasound, the probability of ovulation occurring on the day of or day after the Mira LH surge is close to 80%. Thus the estimated day of ovulation (EDO = Day 0) used in this study was the day of the Mira LH surge.

### Statistical analysis

Demographic, clinical and hormonal characteristics were analyzed using R software (R version 4.3.2, The R Foundation for Statistical Computing). Continuous variables (age, BMI, cycle length, follicular/luteal phase length) were described with means and standard deviations, with differences between groups calculated with ANOVA and post-hoc pairwise comparisons (significance level *p* < 0.05, with post-hoc Bonferroni corrections). Discrete variables (gravida, parity) were described with median and interquartile intervals and categorical variables (ethnicity, education) were described as percentages; group differences were calculated with non-parametric Mann-Whitney U test. Missing data was excluded.

**Fig. 3:**
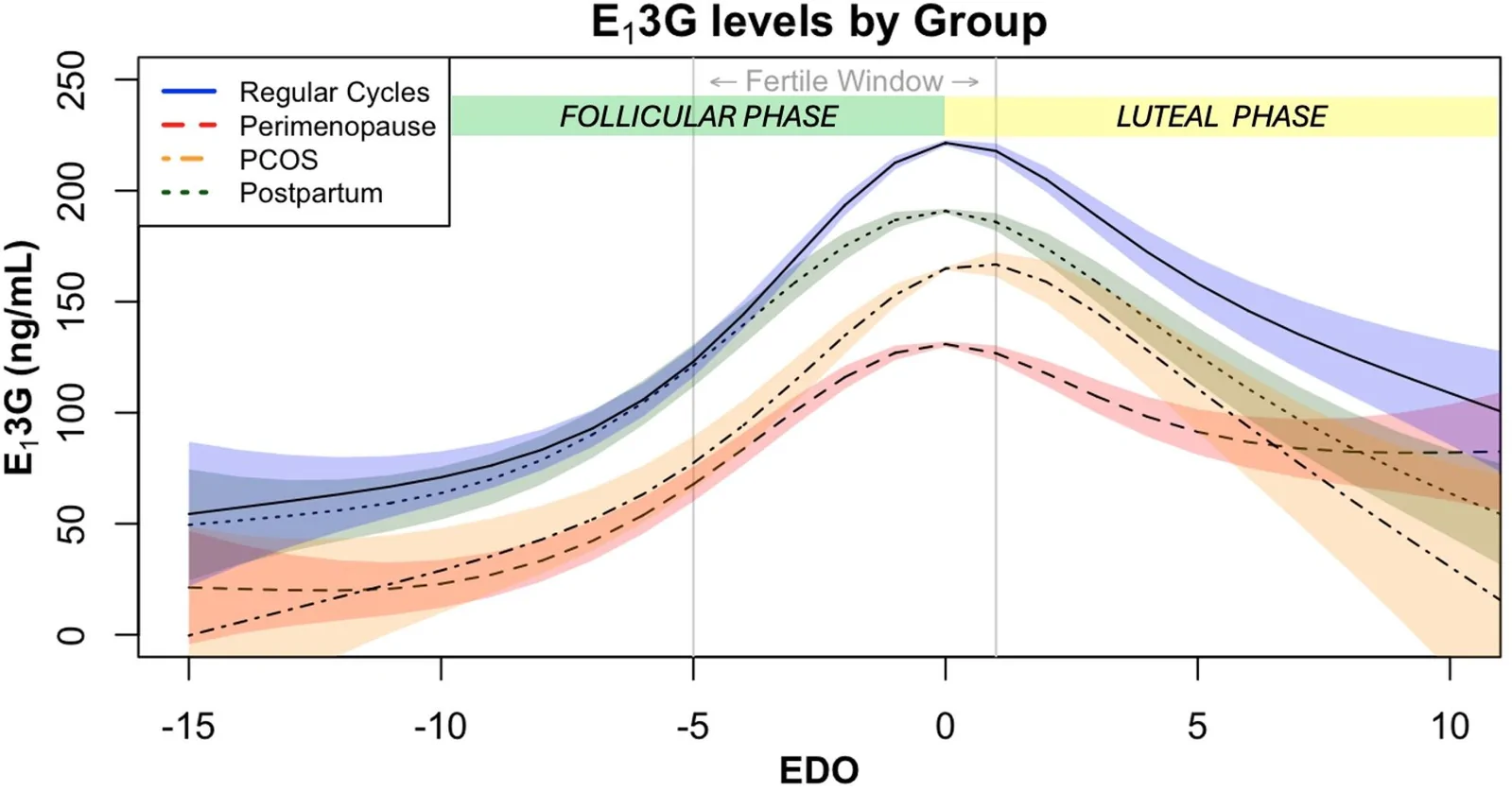
E_1_3G hormone levels stratified by reproductive category (Regular Cycles *n*=10, Perimenopause *n*=13, PMOS, *n*=10, Postpartum *n*=24). Estrone-3-glucuronide (E_1_3G) hormone values (ng/mL) plotted against the estimated day of ovulation (EDO, EDO=0 is the day of the Mira LH surge), with four curves based on the four groups shown in the legend (regular cycles, perimenopause, PMOS, postpartum). The fertile window is delineated by the two grey bars from days -5 to +1 relative to the EDO. The follicular and luteal phases are marked in bands (negative days are the follicular phase and positive days are the luteal phase)

**Fig. 4:**
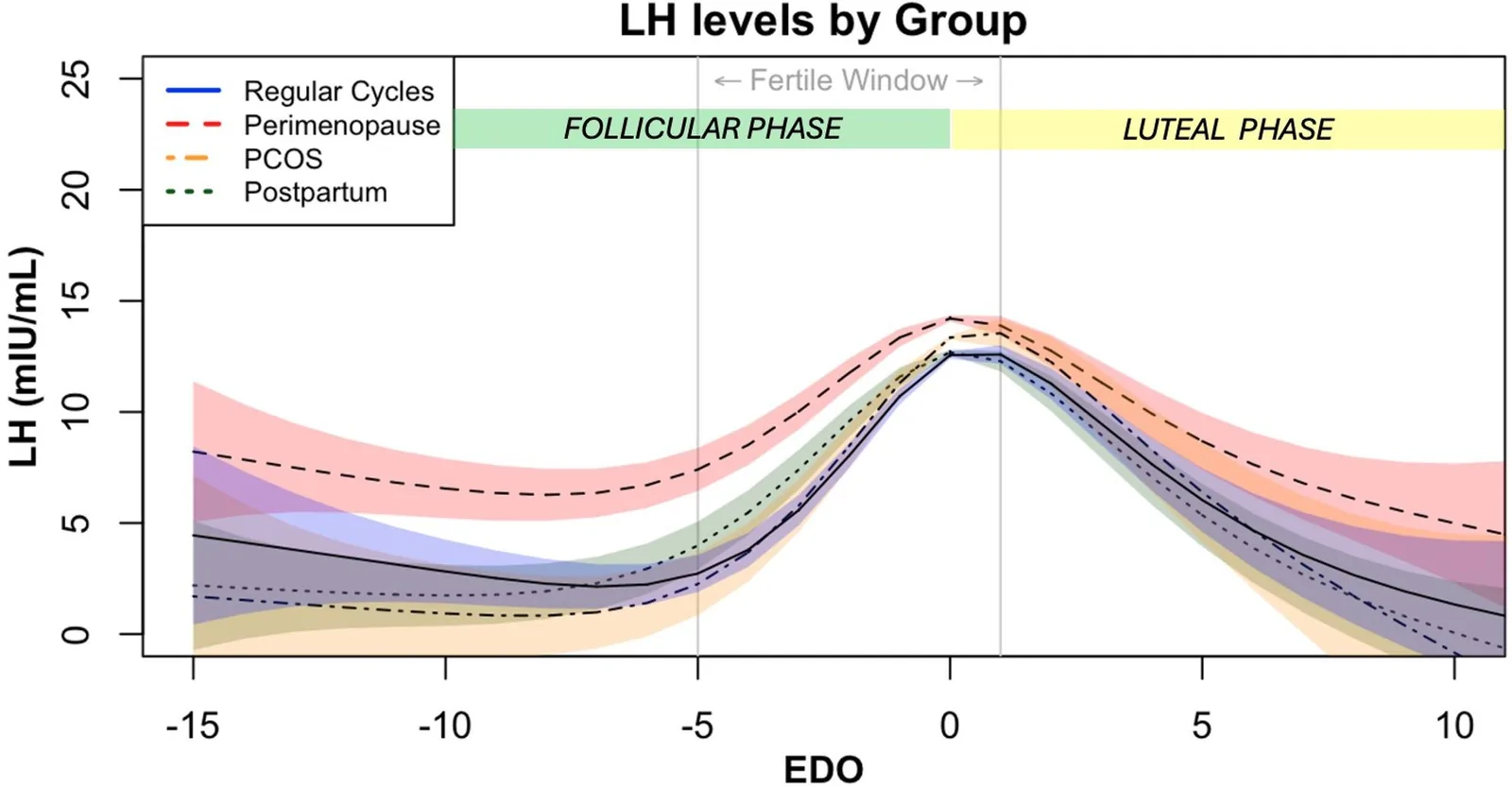
LH hormone levels stratified by reproductive category (Regular Cycles *n*=10, Perimenopause *n*=13, PMOS, *n*=10, Postpartum *n*=24). Luteinizing hormone (LH) hormone values (mIU/mL) plotted against the estimated day of ovulation (EDO, EDO=0 is the day of the Mira LH surge), with four curves based on the four groups shown in the legend (regular cycles, perimenopause, PMOS, postpartum). The fertile window is delineated by the two grey bars from days -5 to +1 relative to the EDO. The follicular and luteal phases are marked in bands (negative days are the follicular phase and positive days are the luteal phase)

**Fig. 5:**
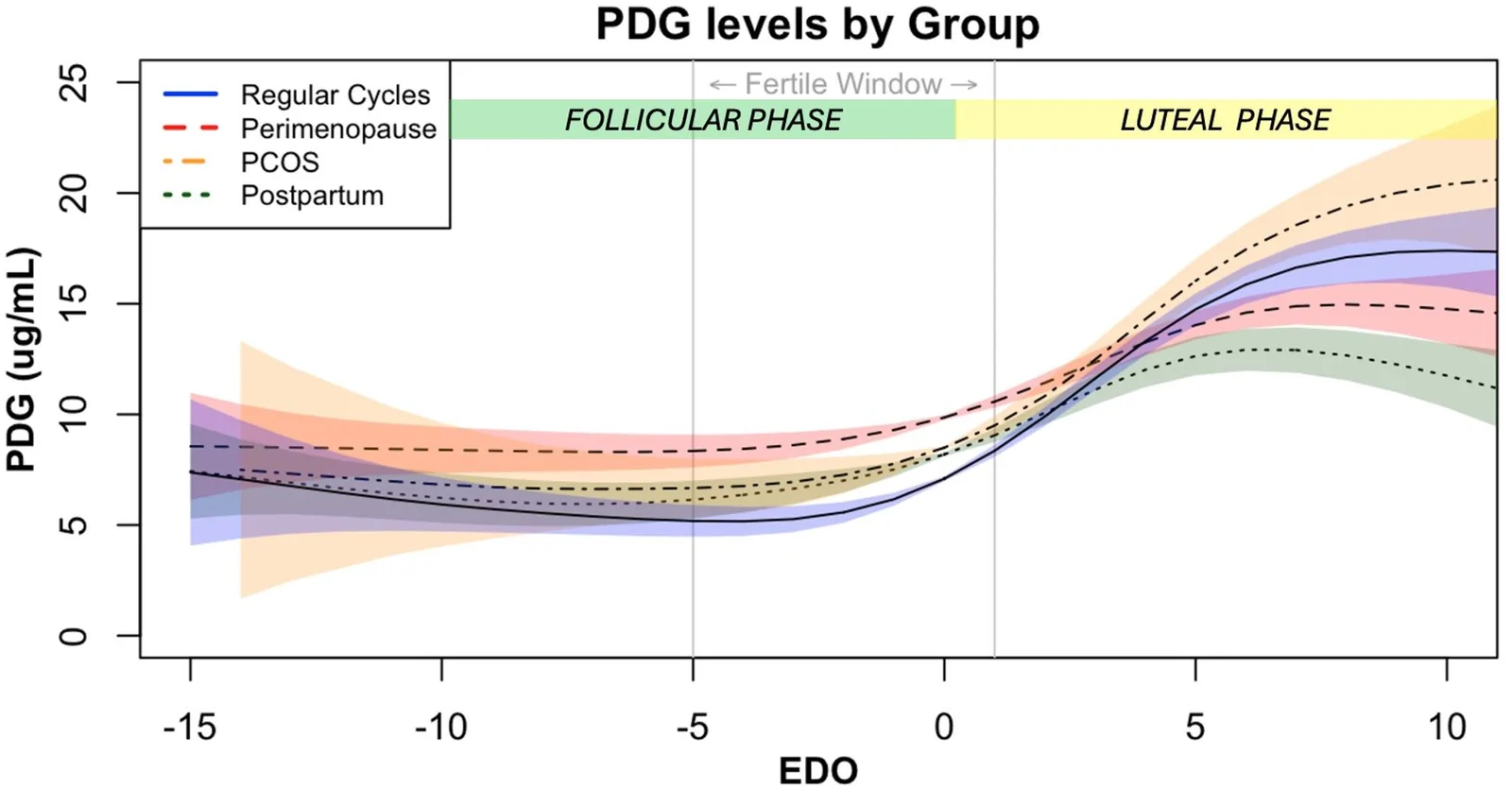
PDG hormone levels stratified by reproductive category (Regular Cycles *n*=10, Perimenopause *n*=13, PMOS, *n*=10, Postpartum *n*=24). Pregnanediol glucuronide (PDG) hormone values (ug/mL) plotted against the estimated day of ovulation (EDO, EDO=0 is the day of the Mira LH surge), with four curves based on the four groups shown in the legend (regular cycles, perimenopause, PMOS, postpartum). The fertile window is delineated by the two grey bars from days -5 to +1 relative to the EDO. The follicular and luteal phases are marked in bands (negative days are the follicular phase and positive days are the luteal phase)

**Fig. 6:**
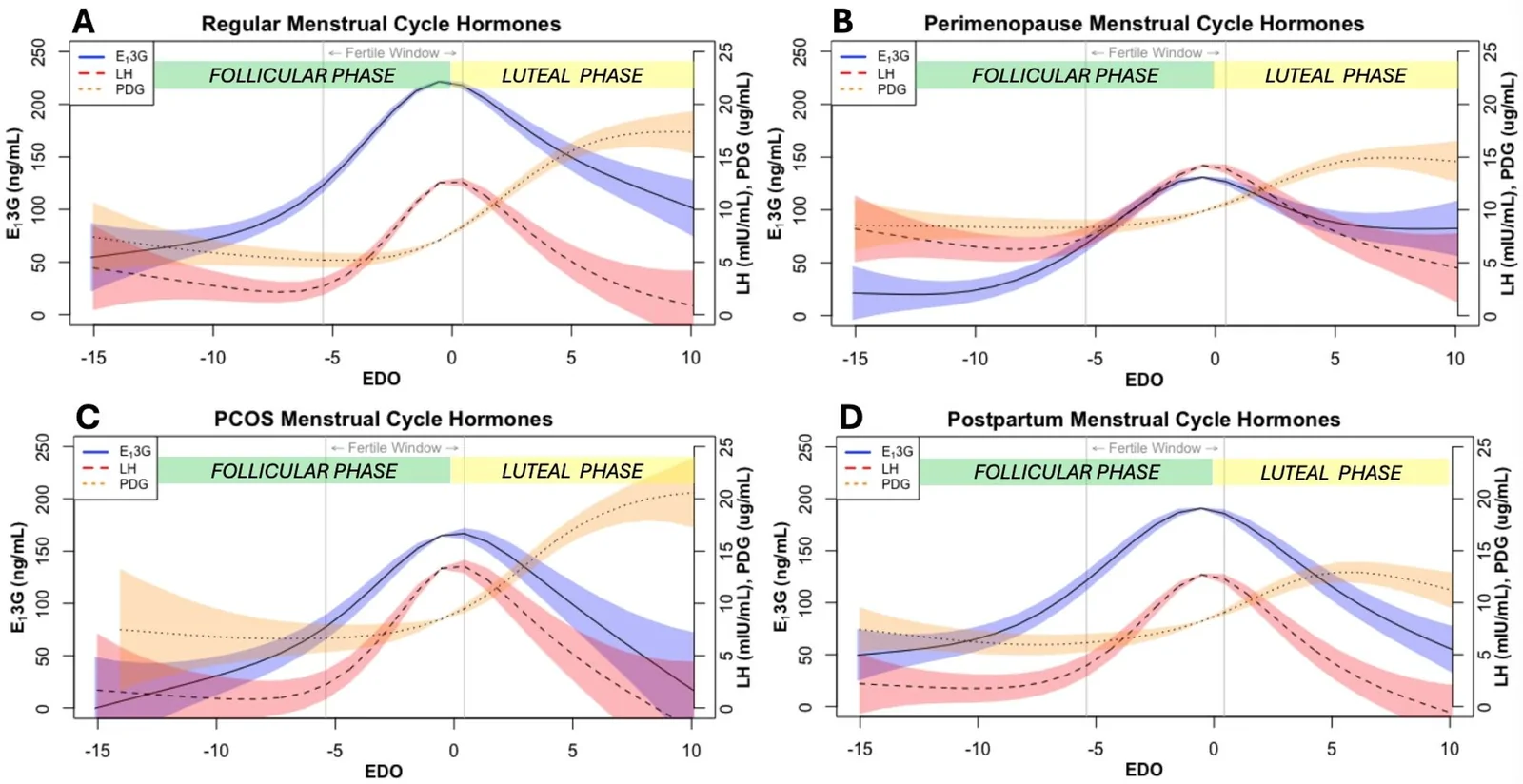
Hormone profiles by reproductive category (Regular Cycles *n*=10, Perimenopause *n*=13, PMOS, *n*=10, Postpartum *n*=24). All three hormones (E_1_3G, LH and PDG) are plotted against the estimated day of ovulation (EDO, EDO=0 is the day of the Mira LH surge), separated for each of the four groups (**A**) regular cycles, (**B**) perimenopause, (**C**) PMOS, (**D**) postpartum. The fertile window is delineated by the two grey bars from days -5 to +1 relative to the EDO. The follicular and luteal phases are marked in bands (negative days are the follicular phase and positive days are the luteal phase)

The theoretical fertile window used for the statistical modeling was based on previously established criteria being 6 days up to and including the day of ovulation (EDO) [29]. Hormonal data were analyzed using additive mixed models, establishing a best fit based on smoothing splines for the hormones. Group differences were assessed using ANOVA for the effect of the groups, while adjusting for random effects of individual women and individual cycles (significance level, *p* < 0.05). Significant differences between groups can also be assessed qualitatively by non-overlapping standard error bands on the model plots (Figs. 3, 4, 5 and 6). In addition to the hormone profile modeling that involved smoothing the data, we also calculated differences between groups with ANOVA for mean hormone values at the beginning of the fertile window (5 days before peak LH), on the peak LH day, and in the luteal phase (5 days after the peak LH day), again with significance levels at *p* < 0.05.

## Results

### Participants and demographics

An initial group of 141 women consented to be contacted for the study (Fig. 1). There were 65 participants who contributed sufficient data to be included in the study. Three of these participants withdrew their consent during the study and 5 participants were part of a chemotherapy menstrual cycle tracking group that was analyzed separately. This left a total of 57 participants contributing a total of 444 cycles of data, with an average of 11.6 days of Mira testing per cycle, summarized in Fig. 1 and Table 1. After 6 transition cycles postpartum, there were 10 women who overlapped with other groups: 9 women had regular cycles after the postpartum transition, and 1 woman was in early perimenopause after the postpartum transition. Some of the postpartum participants (20 of the 24 in this study) were previously analyzed in more detail [4]. Breastfeeding status was available for 20 of 24 postpartum participants, 5 were partial breastfeeding and 15 were fully breastfeeding at entry in the study, but the transition from full to partial breastfeeding was not tracked in detail. Of the perimenopause participants, 9 were in early perimenopause and 5 were in late perimenopause (based on STRAW criteria cycle variability, FSH was not measured).

**Table 1:** Demographic and clinical characteristics

Characteristics	Regular Cycles (*n* = 10 + 8)	Postpartum (*n* = 24)	Perimenopause (*n* = 13 + 1)	PMOS (*n* = 10)
Age	32.3 ± 4.2	32.9 ± 5.1	46.2 ± 2.9	30.2 ± 5.0
Gravida	2.5 (1.25-5)	3.5 (2–5)	5.5 (4.25–9.5)	1 (0-3.25)
Parity	2 (1–3)	2 (2–4)	4 (3-6.25)	1 (0–1.0)
BMI	24.5 ± 3.7	25.8 ± 6.6	27.6 ± 7.9	29.0 ± 6.3
Race				
* White*	17 (94.4%)	20 (83.3%)	14 (100%)	9 (90%)
* Black*		2 (8.3%)		
* Hispanic*	1 (5.6%)	2 (8.3%)		
* Other*				1 (10%)
Highest Education				
* High School*	2 (11.1%)	2 (8.3%)	3 (21.4%)	2 (20%)
* Undergraduate*	9 (50%)	11 (45.8%)	7 (50%)	3 (30%)
* Graduate*	5 (27.8%)	7 (29.2%)	2 (14.3%)	4 (40%)
* Unknown*	2 (11.1%)	4 (16.7%)	2 (14.3%)	1 (10%)
Perimenopause				
* Early*			8 (71.4%)	
* Late*			4 (28.6%)	

Data are mean +/- standard deviation for continuous variables (age, BMI), median (interquartile range) for discrete variables (gravida, parity), and n (%) for categorical variables (Race, Education, STRAW perimenopause criteria). Samples for each group include overlapping numbers +n for those cycles after 6 transition cycles postpartum, e.g. 8 participants with regular cycles after 6 transition cycles postpartum

*BMI* body mass index

The population in this study was homogeneous (Table 1) - predominantly white, university educated women - not unlike previous studies of women who use hormone fertility monitors [30]. The perimenopause women were significantly older than the other 3 groups (*p* < 0.001). Perimenopause women had higher parity than the other 3 groups (Mann-Whitney U test *p*-values were all < 0.005). Women with PMOS had the lowest number of pregnancies and parity compared to all 3 groups (Table 1).

### Cycle characteristics

Cycle length was significantly longer for the postpartum transition cycles than for regular cycles (*p* = 0.01), but the other 3 groups did not significantly differ in cycle length (Table 2). Follicular phase length was longer (i.e. later peak day) in postpartum transition cycles, as previously described [4], than regular cycles (*p* < 0.001) and perimenopause women (*p* = 0.001), but not significantly different from PMOS women. Luteal phase length was shorter in postpartum women than all the other groups (*p* < 0.001).

**Table 2:** Cycle Parameters

Characteristics	Regular Cycles (*n* = 10 + 8)	Postpartum* (*n* = 24)	Perimenopause (*n* = 13 + 1)	PMOS (*n* = 10 + 3)
Cycle length	28.7 ± 3.3^a^	33.1 ± 22.9^a^	29.5 ± 7.3	30.5 ± 5.7
Follicular phase length	15.3 ± 3.1^b^	22.4 ± 22.8^b, c^	16.6 ± 6.8^c^	17.8 ± 6.1
Luteal phase length	13.4 ± 1.6^d^	10.6 ± 2.7^d, e,f^	12.9 ± 2.8^e^	12.8 ± 2.1^f^

Data are mean +/- standard deviation

*LH* luteinizing hormone

Significant paired-samples differences are shown by superscript letters: ^a^*p*=0.01, ^b^*p*<0.001, ^c^*p*=0.001, ^d,e,f^*p*<0.001

*Postpartum cycle parameters exclude postpartum amenorrhea

### Hormone profile modeling

Hormone profiles (E_1_3G, LH and PDG) were modelled for each group with additive effects for each reproductive category and random effects per woman and per cycle (Figs. 3, 4, 5 and 6). Raw data with threshold values for each hormone on days − 5, 0 and + 5 are shown in Table 3. In Figs. 3, 4 and 5, non-overlapping standard error bands indicate a significant difference between the groups. The non-overlapping standard error bands (Figs. 3, 4, 5 and 6) and the calculation of significant differences between groups for the raw data (Table 3) showed a coherence in demonstrating group differences in hormone levels at the three time points in the cycle.

**Table 3:**
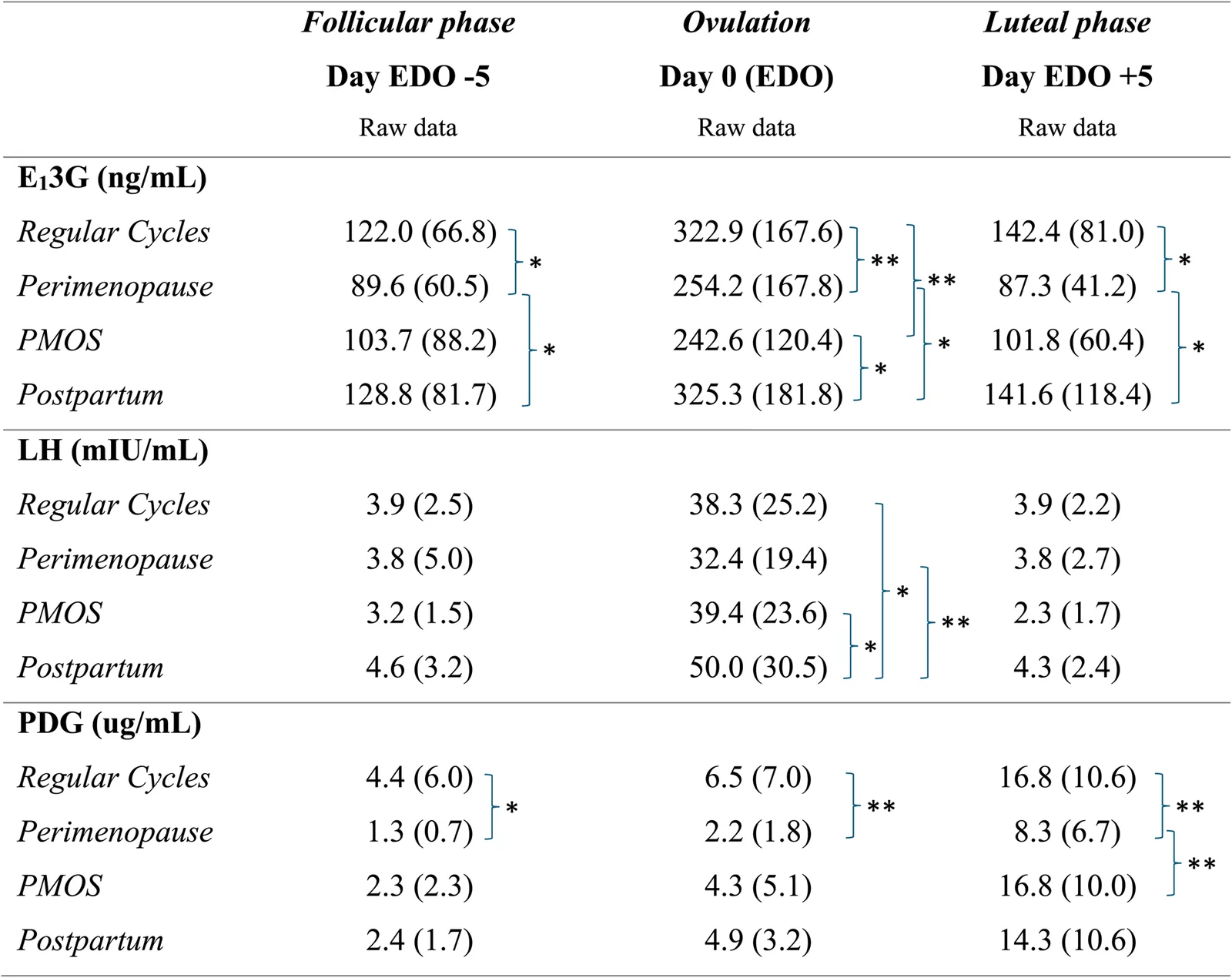
Raw data hormone mean values (SD) for day − 5, 0 (EDO) and + 5, stratified by group

Data points are at 3 days in the cycle, EDO -5, which is the beginning of the fertile window in the follicular phase, day=0 (EDO) which is the LH peak day, and EDO +5 which is in the luteal phase

Significant differences in raw data are marked by * for *p*<0.05 and ** for *p*<0.005. Differences in model intercepts are identified by non-overlapping standard error shading bands in Figs. 2, 3, 4, 5

*E*_*1*_*3G* estrone-3-glucuronide, *LH* luteinizing hormone, *PDG* pregnanediol glucuronide

There is a significant difference in E_1_3G levels among all reproductive categories, with women in regular cycles having higher levels overall, especially in the fertile window and the luteal phase. The perimenopause group reflected an overall hypoestrogenic state, with less of a decline in estrogen through the luteal phase than the other groups. The PMOS group also had a relatively hypoestrogenic state compared to the postpartum and regular cycles group in the follicular phase and fertile window (Fig. 3).

In the LH model (Fig. 4), the perimenopause group had significantly higher LH values in the follicular phase and fertile window, even though the raw data shows that the Postpartum group clearly has higher LH values (Table 3). The LH surge is concentrated in all groups around the EDO (day 0).

Urinary progesterone metabolites (PDG) clearly showed the luteal phase shift. The PMOS group had the highest PDG levels, overlapping with the regular cycles group, significantly higher than both postpartum and perimenopause. The postpartum group had the lowest PDG in the model (Fig. 5) but the perimenopause group had the lowest PDG in the raw data (Table 3).

A summary of all three hormone profiles stratified by group is shown in Fig. 6, showing the group differences described above, demonstrating the hypoestrogenic state of the PMOS and perimenopause groups and the hypoprogestogenic state of perimenopause and postpartum groups.

## Discussion

### Principal findings and results

The fertile window delineated by urine hormone metabolites using the Mira monitor varies depending on a woman’s reproductive category. Women are already using these technologies on their own and will likely seek to integrate this with advice from their clinicians. With more validation studies on FemTech devices, clinicians may consider turning to evaluating personalized daily urinary hormone patterns rather than infrequent serum hormone checks that are difficult to extrapolate into the bigger picture of each menstrual cycle. Specifically, urine sampling of hormones can allow for averaging of hormone levels over many days in the context of pulsatile hormone fluctuations (e.g. for E_1_3G and PDG).

In PMOS, fertile window hormonal variability is not surprising, given that there is known estradiol [20] and LH [21] abnormalities to explain the cycle irregularities. In postpartum and perimenopause transitions [25], hormone changes are present as fertility returns postpartum or wanes towards menopause. The present study demonstrated differences in the fertile window in women with perimenopause, relative to the other three groups. Our data clearly show hypoestrogenism in perimenopause, which has been well described [31], but of interest, the luteal E_1_3G plateau (Fig. 3) that we observed in perimenopause may reflect previous findings of loop-out-of-phase follicular development in the perimenopause transition [32]. Levels of LH were high across the cycle in this group (Fig. 4), with previous data showing multiple LH rises occur in this group [25]. Low luteal PDG (Fig. 5) in the perimenopause may reflect underlying abnormal luteinization processes [33].

In the postpartum group, which has been previously analyzed [4, 25], higher raw LH values (Table 3) are likely due to a decreased sensitivity of the ovary to LH (higher values are required to trigger ovulation). In the model (Fig. 4), this was reflected in the earlier LH rise in the fertile window compared to the regular cycles and PMOS group. The postpartum group had relatively similar E_1_3G values compared to the regularly cycling group, which were higher than the PMOS and perimenopause groups for most of the cycle, reflecting their similarities in follicular E_1_3G to women in regular cycles.

The PMOS group had lower overall E_1_3G and a higher luteal PDG levels, with minimal differences in LH secretion. If follicular development in women with PMOS were stimulated with medications, it would be possible to track and individualize response to fertility treatments in PMOS using urinary hormones.

The typical “textbook” menstrual cycle is the result of taking average hormone results and plotting them on a curve. An alternative to this would be individualizing assessment of the menstrual cycle by tracking urinary hormones to identify the group and individual hormonal variability [14, 34–36]. Presently, applying a personalized fertile window has been left to smartphone Apps that are often inaccurate [37], or industry-developed tools that have yet been validated [1] or have proprietary algorithms that are not open-source. Clinicians should be aware of which tools available to patients are validated to help their patients track their reproductive hormones accurately. The results of this study were not compared to the proprietary Mira algorithm used in the device’s App, but a comparison to their algorithm could be considered in the future.

In the current study, we have demonstrated the importance of factoring a woman’s reproductive category (e.g. regular cycles, postpartum, PMOS, perimenopause; of course many other categories could be added to this) in delineating the fertile window, since thresholds of E_1_3G, LH and PDG were different in these four different groups (Figs. 3, 4, 5 and 6, Table 3). In the future, a woman’s individual hormone pattern can help to identify her own personalized fertile window that becomes more precise as more cycles are added to the predictive model. This predictive modeling is not only an educational opportunity to increase menstrual and ovulation health literacy using these tools [38], but also a collaborative opportunity for clinicians and their patients to identify abnormalities that could help track response to treatment (e.g. for PMOS treatment).

With a larger sample size of women with PMOS, it may have been possible to see significant differences in cycle length, later peak days and shorter luteal phases compared to women with regular cycles. Similar non-significant signals were found in perimenopause, and it is possible that perimenopause and PMOS may follow similar patterns. It should also be noted that most of our perimenopause participants were in early perimenopause, which may skew the cycle parameters which a larger sample size with more women in late perimenopause would clarify. Specifically, cycle lengths in this smaller sample are likely shorter than a group that included more women in late perimenopause with longer cycles. In the future, with the use of a Mira FSH test, these hormone results may help in predicting menopause. The developers of the ClearBlue Fertility Monitor have also proposed a new FSH test for predicting menopause [39].

The clinical relevance of characterising the fertile window in perimenopause is twofold. First, perimenopausal women who wish to avoid pregnancy have to deal with irregular cycles, multiple LH rises and out-of-phase follicular activity previously described [32] which make conventional calendar- or symptom-based fertile-window estimates unreliable; quantitative urinary hormone data offer an individualized way to identify potentially ovulatory events for family planning decision-making at a life stage when unintended pregnancy still occurs. Second, the perimenopause-specific hormone patterns we described — relative hypoestrogenism, persistently elevated LH, low luteal PDG and an atypical luteal E_1_3G plateau (Figs. 3, 4, 5 and 6, Table 3) — may be clinically meaningful - they could provide an objective, at-home tool for tracking the underlying hormonal changes of perimenopause symptoms.

### Strengths and limitations

Since our study’s primary objective was demonstrating the feasibility to detect significant differences in the fertile window between the four reproductive categories, the current study will be used to calculate the power for future studies with larger sample sizes to help identify more subtle differences between the groups, for example, to detect pairwise differences in E_1_3G between any two of the four groups at a moderate effect size (Cohen’s *d* = 0.5, power 0.8 and alpha 0.05), approximately 64 participants per group would be required; with Bonferroni correction for the six pairwise comparisons among the four groups, 99 participants would be needed. The main limitation was the lack of ultrasound-confirmed ovulation in the present study, but since the Mira monitor has recently been validated with ultrasound [40], this increases confidence in our findings. Multiple ultrasound-based validation studies have shown that urine PDG [41] and serum progesterone [42, 43] can confirm ovulation, yet validation of the Mira monitor now improves the interpretation of these results and justifies expansion to reach broader groups of women.

The sample was relatively homogenous, so future studies should focus on recruiting women with more diverse backgrounds to evaluate the role of ethnic, socioeconomic or other sources of diversity that may lead to greater personalization of the fertile window. To improve generalizability, future studies should broaden recruitment venues and reach out to participants who have not previously used fertility monitors. The cost of the monitor and test wands are barriers for using these tools, but the economic benefits of urine metabolite testing at-home could lead to system-wide reduction in health care costs (e.g. with associated reduction serum hormone testing) that may lead health policy makers to consider advocating for insurance plans to cover these new testing tools just as diabetic supplies are now ubiquitously covered. Although we corrected for covariates in our model including individual women and cycle as random effects, and there were significant differences in the model and in the raw data, which can be explained by the fact that the model highlights the specific effects of the group, rather than just the raw averages. Future studies should also consider differences in raw vs. modelled data, to find the most appropriate way to represent group vs. individual effects.

Follow-up studies should include objective criteria in categorizing both PMOS and perimenopause groups, since the present study was based on self-reporting for both groups. Formal clinical diagnoses stratified by consensus criteria need to be included in larger studies, since different sub-groups could then be evaluated, and would help to avoid misclassifications that could have existed in our dataset with self-report. Indeed, PMOS studies evaluating hormone patterns have shown different hormone results depending on the lean vs. obese PMOS sub-groups [44, 45]. Another important consideration would be to collect more detailed information about breastfeeding status, including partial (with amounts) vs. full breastfeeding, and a prospective weaning schedule which could further clarify the impact of lactation on the hormones of the cycle. Lastly, the economic impact of at-home urine testing versus serum hormone panels should be considered in more detail in future studies, since a full cycle of daily urine hormone testing showing the full menstrual cycle hormone profiles would be roughly the same cost as two days of serum hormone sampling.

## Conclusions

Personalization of the fertile window is not a new concept [46], but the integration of tools like urine fertility monitors with smartphone Apps [1, 30] require evidence-based validation for clinicians to be confident in recommending them to patients. Validating new technology for at-home menstrual cycle monitoring should be a high priority for clinicians and health authorities to contribute to increasing menstrual health literacy and improved integration with women’s fertility goals [38]. These monitoring tools should be validated against the current gold-standard of ultrasound-defined ovulation, as well as correlated with serum hormone values which is the current standard of practice for menstrual cycle monitoring [3]. This validation will then allow us to identify specific hormone thresholds for the fertile window, as well as compare the Mira Monitor with other menstrual cycle monitoring tools.

## Data Availability

Individual participant data that underlie results after deidentification could be provided to researchers to achieve aims in a methodologically sound proposal approved by an independent review committee up to 36 months after article publication.

## Abbreviations

CBFM: ClearBlue Fertility Monitor
E_1_3G: Estrone-3-glucuronide
EDO: Estimated day of ovulation
LH: Luteinizing hormone
PCOS/PMOS: Polycystic ovarian syndrome/polyendocrine metabolic ovarian syndrome
PDG: Pregnanediol glucuronide
STRAW: Stages of Reproductive Aging Workshop
